# Beyond prompt engineering: the associations among creative self-efficacy, flow state, learner engagement, and psychological ownership in GenAI-assisted composition

**DOI:** 10.3389/fpsyg.2026.1823846

**Published:** 2026-05-29

**Authors:** Tianyu Tong

**Affiliations:** Graduate School, Anyang University, Anyang, Gyeonggi-do, Republic of Korea

**Keywords:** creative self-efficacy, flow state, generative AI, human-AI co-creation, learner engagement, music education, psychological ownership, text-to-music

## Abstract

**Introduction:**

The integration of generative artificial intelligence (GenAI) into music education markedly lowers technical barriers for predominantly novice composers, but also raises concerns about a potential erosion of human creative agency. When learners rely on text prompts to produce music with minimal subsequent involvement, they may fail to develop a sense of psychological ownership over AI-assisted creations.

**Method:**

Drawing on the Theory of Psychological Ownership, this study examined the cognitive, affective, and behavioral processes through which perceived GenAI support relates to students’ psychological ownership. Survey data were collected from 355 non–music-major undergraduates enrolled in a GenAI-assisted composition course that explicitly required iterative post-generation refinement of AI outputs. Structural equation modeling with bias-corrected bootstrapping was used to test a serial mediation model.

**Results and discussion:**

The results showed that perceived GenAI support was positively and significantly associated with psychological ownership, and that this relationship operated through a sequential pathway involving creative self-efficacy, flow state, and learner engagement. These findings suggest that GenAI does not inherently alienate learners; when positioned as a cognitive scaffold within a human-in-the-loop design, it is associated with creative confidence, optimal immersion, and active investment of effort. The study highlights the importance of deliberately incorporating productive friction into AI-supported learning activities to elicit an “IKEA effect,” thereby transforming algorithmically generated material into personally appropriated creative artifacts.

## 1 Introduction

For decades, formal music composition has remained a relatively exclusive domain, shaped by steep learning curves, extensive theoretical training, and the often prohibitive financial costs of specialized instruction (Collins, 2005; Elpus and Abril, 2011; Hickey, 2012). For students without prior musical training, aspirations to compose original works have frequently been curtailed by these formidable barriers to entry. The recent emergence of generative AI (GenAI), particularly text-to-music models, has begun to democratize this creative process (Gianet et al., 2024; Väkevä and Partti, 2025). By enabling novices to bypass years of technical training and to produce high-fidelity musical artefacts through simple natural language prompts, these tools offer unprecedented instructional scaffolding. Yet, while GenAI substantially lowers barriers to accessibility, it simultaneously introduces a critical pedagogical dilemma: the tension between technological assistance and cognitive substitution (Baird and Maruping, 2021). When the AI assumes most of the cognitive and technical workload, learners risk being repositioned from active creators to passive consumers or mere “prompt engineers.” This displacement raises serious concerns regarding the erosion of human agency in the creative process (Epstein et al., 2020; Wu et al., 2025). In particular, if a student can generate a complete track within seconds, it becomes questionable whether they can still develop a genuine sense of psychological ownership—the cognitive and affective state in which individuals feel that a creative artefact is truly “theirs” rather than the machine’s (Avey et al., 2009; Pierce et al., 2001).

Fostering psychological ownership is not a purely theoretical issue; it is a key driver of sustained learning engagement and the development of creative identity (Katz and Assor, 2007; Pierce et al., 2001). If students feel no personal attachment to AI-generated outputs, the core educational value of a composition course is fundamentally undermined. Paradoxically, despite the centrality of this psychological attachment in GenAI-assisted learning, existing educational technology research has paid limited attention to the learner’s internal experience. Prior work has predominantly examined either the technological acceptance of such tools—often through frameworks like the Technology Acceptance Model (TAM), focusing on perceived usefulness and ease of use—or the objective acoustic quality of AI-generated products (Crompton and Burke, 2023; Strzelecki, 2024). As a result, a substantial gap remains in opening the “black box” of human–AI co-creation, particularly with respect to how learners cognitively and affectively appropriate machine-generated drafts as their own (Ouyang and Jiao, 2021; Weisz et al., 2023). In highly subjective and expressive domains such as music composition, the impact of GenAI support is theoretically contested: it is far from clear whether extensive technological scaffolding diminishes learners’ creative agency, or whether it can, under certain psychological conditions, cultivate a higher-order sense of ownership (Amabile and Pratt, 2016).

The complex chain linking perceived AI support to learners’ eventual appropriation of the co-created product has not yet been empirically mapped, leaving a critical blind spot in contemporary educational technology research. To address this gap, the present study draws on the Theory of Psychological Ownership (TPO) as its primary conceptual lens (Avey et al., 2009; Pierce et al., 2001). TPO posits that one principal route to experiencing ownership is the “investment of self,” a mechanism closely aligned with the IKEA effect in behavioral economics, whereby individuals ascribe greater value to products they have actively labored to create (Norton et al., 2012). Applied to GenAI-assisted music composition, we conceptualize the development of psychological ownership as a sequential cognitive, affective, and behavioral process.

We propose that the instructional scaffolding afforded by GenAI (perceived GenAI support) first reduces technical barriers and strengthens learners’ belief in their own creative capabilities—creative self-efficacy (Tierney and Farmer, 2002; H1). This enhanced confidence, in turn, is associated with a shift in the composition task from a daunting challenge into an optimally engaging experience, facilitating a flow state (Csikszentmihalyi, 2014; H2). Rather than passively accepting the AI’s initial outputs, learners are thus motivated to invest effort in iteratively refining and editing the drafts. We argue that this behavioral shift (learning engagement) is jointly driven by robust creative self-efficacy, which enables learners to tackle refinement tasks (Bandura, 1997; Linnenbrink and Pintrich, 2003; H3), and by the intrinsically rewarding nature of flow, which sustains their continued effort (Shernoff et al., 2014; H4). Ultimately, psychological ownership is associated with two pathways. First, the affective resonance and intense concentration associated with flow foster a deep emotional connection to the music (Pierce et al., 2001; H5). Second, the behavioral manifestation of the IKEA effect—the substantial investment of time and cognitive resources during the refinement process (learning engagement)—constitutes an “investment of self” that is linked to learners’ sense of ownership over the musical artefact (Avey et al., 2009; Norton et al., 2012; H6).

**Figure 1:**
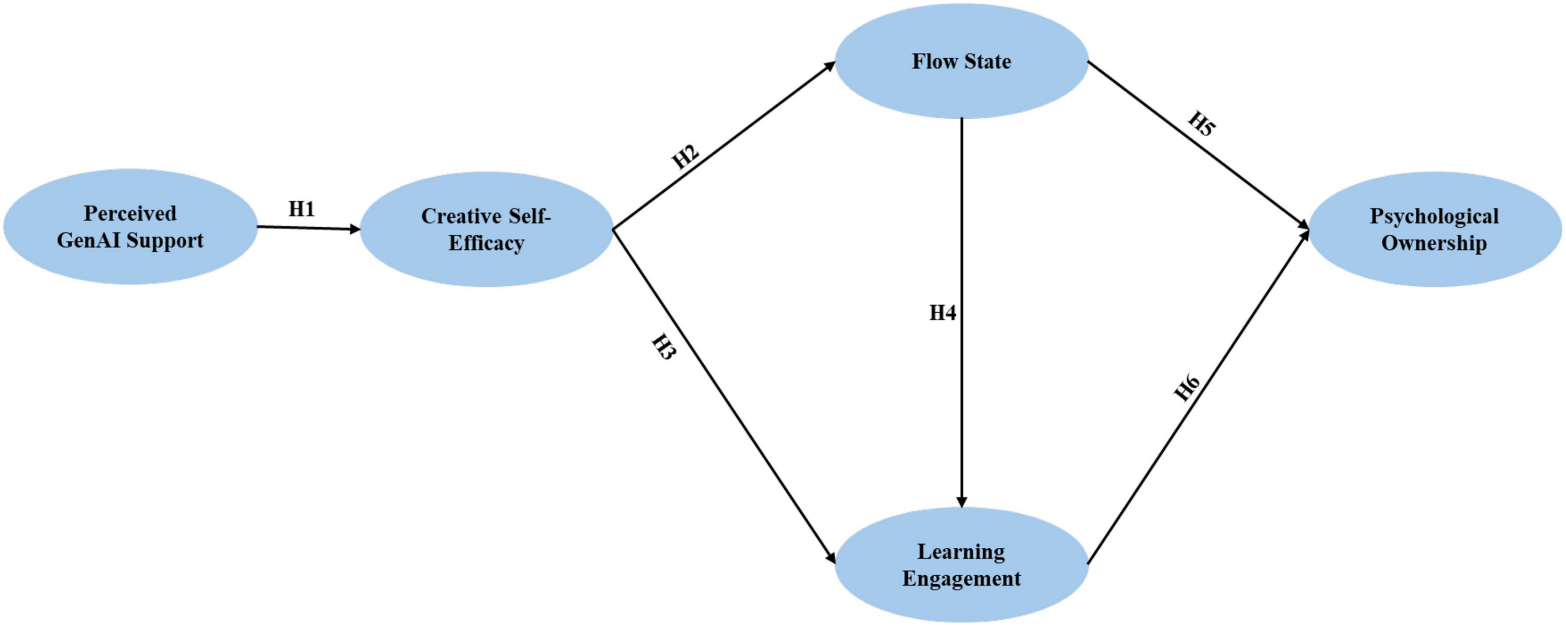
Hypothesized structural equation model for perceived GenAI support, creative self-efficacy, flow state, learning engagement, and psychological ownership.

Integrating these hypotheses, the proposed serial mediation model (Figure 1) delineates both the direct and indirect pathways through which technological scaffolding may translate into psychological ownership in GenAI-assisted composition. Accordingly, this study addresses the following research questions (RQs):

- RQ1: Is there a significant relationship between perceived GenAI support, creative self-efficacy, flow state, learning engagement, and psychological ownership in GenAI-assisted music composition?
- RQ2: To what extent do creative self-efficacy, flow state, and learning engagement serially mediate the relationship between perceived GenAI support and learners’ psychological ownership?

## 2 Literature review

### 2.1 GenAI in music education and the theory of psychological ownership

As GenAI permeates multimodal domains, educational technology research has increasingly reconceptualized the role of AI in music education from a merely assistive tool to an intelligent collaborator with independent generative capabilities (Väkevä and Partti, 2025). Traditional computer-assisted music instruction has typically focused on digital notation, sequencing, or audio editing (Carnovalini et al., 2025), thereby leaving the full burden of conceptual design and technical execution on the learner. By contrast, GenAI models—particularly text-to-music systems—fundamentally reshape the creative workflow by shifting learners’ cognitive load from low-level note arrangement and harmonic construction to higher-level tasks such as conceptual design and prompt formulation (Cheng, 2025). Recent empirical work suggests that this human–AI collaborative paradigm can substantially influence learners’ creative performance (Wang et al., 2025). For example, Zhuang and Li (2025) reported that GenAI-supported collaborative composition significantly broadened students’ creative boundaries, while Li et al. (2025) showed that designing with AI facilitated pre-service music teachers’ internalization of technological pedagogical content knowledge. At the same time, these studies caution that when AI assumes most of the generative work, the resulting asymmetry of control between human and machine may fundamentally alter how learners psychologically relate to the final creative artefact (Merchán Sánchez-Jara et al., 2024).

To unpack the psychological mechanisms underlying this new form of human–computer co-creation, the present study adopts the TPO as its analytical framework. Originating in organizational behavior, TPO explains why individuals develop a sense of possession—a feeling that “this is mine”—toward targets they do not legally own (Pierce et al., 2001). Drawing on the seminal work of Pierce et al. (2001) and Avey et al. (2009), psychological ownership is understood to arise through three primary routes. The first is control over the target, referring to the individual’s capacity to influence and shape the target’s development. The second is intimate knowing, whereby continuous interaction fosters deep cognitive and affective familiarity with the target’s attributes (Avey et al., 2009; Pierce et al., 2001). The third is investment of self, which involves the infusion of one’s time, effort, emotion, and intellectual labor into the target (Norton et al., 2012; Pierce et al., 2001). In digital learning and human–computer interaction, these three routes are considered prerequisites for learners to internalize external knowledge or AI-generated artefacts as part of their own psychological domain (Füller et al., 2009; Ouyang and Jiao, 2021).

In the context of GenAI-assisted music composition, these classical routes are both disrupted and reconfigured (Ouyang and Jiao, 2021). Because text-to-music tools can generate initial musical excerpts within seconds, the interaction is characterized by extremely low friction. This immediacy can undermine learners’ initial investment of self and intimate knowing, as little time or effort is required to produce a complete draft (Epstein et al., 2020; Weisz et al., 2023). Accordingly, this study posits that in highly automated generative environments, psychological ownership is unlikely to emerge at the moment of “prompt-and-generate” alone (Baird and Maruping, 2021). Instead, it depends critically on what happens after generation—namely, on learners’ engagement in evaluating, iterating, and refining the AI’s initial output. Only when learners actively participate in these deeper cognitive and behavioral processes can the three TPO mechanisms be reactivated (Weisz et al., 2023). This theoretical lens provides a foundation for examining how creative self-efficacy, flow state, and learning engagement operate as mediating mechanisms that bridge the ownership gap in human–AI co-creation.

### 2.2 The cognitive-affective pathway: AI support, creative self-efficacy, and flow state

Creative self-efficacy (CSE), defined as individuals’ beliefs in their capability to produce novel and useful outcomes, is a critical cognitive antecedent of creative performance (Tierney and Farmer, 2002). In music education, self-efficacy not only predicts academic achievement but also functions as a core psychological resource for mitigating music performance anxiety (Zelenak, 2024). However, conventional music composition is typically characterized by steep learning curves and high technical thresholds (Collins, 2005; Hickey, 2012). Although prior research suggests that computer-assisted tools such as digital audio workstations can enhance students’ musical self-efficacy and creative thinking (Yanan, 2024), these systems still presuppose a basic command of music theory and software operation. As a result, they do not fundamentally alleviate the cognitive demands placed on novices.

According to self-efficacy theory, enactive mastery experiences constitute the most powerful source of efficacy beliefs (Hendricks, 2016). In GenAI-assisted music co-creation, text-to-music tools provide substantial cognitive scaffolding through natural language interaction, substantially reducing the difficulty of technical execution (Gianet et al., 2024; Väkevä and Partti, 2025). This form of support can enable learners without specialized musical training to generate high-quality outputs within a relatively short period of time. Such rapid, low-friction mastery experiences have the potential to reduce initial apprehension toward complex creative tasks and, in turn, to strengthen learners’ confidence in their own creative capabilities. On this basis, we hypothesize that perceived GenAI support positively predicts learners’ creative self-efficacy (H1).

An increase in self-efficacy at the cognitive level also lays an important foundation for deeper affective involvement. Flow is conceptualized as an optimal experiential state characterized by intense concentration, intrinsic enjoyment, and a heightened sense of productivity (O'Neill, 1999). Classical theories of music learning emphasize that flow emerges when there is an appropriate balance between task challenge and perceived skill (Custodero, 2002): when challenge exceeds skill, anxiety is likely; when skill exceeds challenge, boredom tends to occur. Recent studies further indicate a close interplay between self-efficacy and flow in multifaceted music learning environments (Su et al., 2024).

Within the specific context of GenAI-assisted composition, AI tools absorb much of the low-level workload associated with harmonic construction and audio rendering, while learners, supported by GenAI, develop stronger beliefs in their creative capabilities (i.e., heightened CSE). This enhanced CSE can foster a subjective sense of control over the AI collaborator and the broader co-creative process, including higher-order conceptual design and iterative prompt refinement. When learners perceive that their creative capabilities are well matched to the demands of the GenAI-supported task, they are more likely to avoid technology-induced anxiety and to enter an immersive flow state. Accordingly, this study posits that creative self-efficacy positively predicts learners’ flow state (H2).

### 2.3 The behavioral translation and the IKEA effect: learning engagement to ownership

Learning engagement is widely recognized as a key predictor of academic achievement and educational satisfaction (Lane et al., 2021; Wong et al., 2024). It is commonly conceptualized as a multidimensional construct that encompasses learners’ sustained cognitive, emotional, and behavioral involvement in the learning process (Halverson and Graham, 2019). Sustaining such engagement, however, is particularly challenging in digital learning environments heavily mediated by generative AI. Recent work by Fan et al. (2025) cautions that the powerful generative capabilities of GenAI can foster metacognitive complacency: when AI systems are able to produce seemingly polished, high-fidelity outputs instantaneously, learners may be tempted to bypass deeper cognitive processing and meaningful behavioral investment, thereby slipping into a passive, consumption-oriented stance. To counteract this social loafing tendency in human–AI co-creation, learners require strong internal drivers that motivate them to engage in the iterative refinement and editing of AI-generated drafts. It is theoretically crucial to delineate the distinction between flow state and learner engagement within this process. While both constructs involve deep immersion, they operate at different temporal and psychological levels. Grounded in Csikszentmihalyi’s (2014) framework, flow is an acute, ephemeral, and optimal *state* experience—an immersive spark characterized by spontaneous joy and an altered sense of time that occurs *during* the immediate execution of a task. In contrast, learner engagement represents a broader, more deliberate, and sustained *investment* of behavioral, cognitive, and emotional energy across the entire learning cycle (Fredricks et al., 2004). In our human-AI co-creation model, flow acts as the affective catalyst that subsequently relates to the sustained, effortful labor of post-generation refinement.

This study argues that the shift from passive reliance on AI to active engagement in co-creation is associated with a cognitive–affective dual mechanism. Cognitively, creative self-efficacy (CSE) provides learners with the belief that they are capable of handling complex refinement tasks. Drawing on social cognitive theory, high self-efficacy is known to shape the amount of effort individuals invest and their persistence when facing challenges (Bandura, 1997). When learners are confident that they can work effectively with the AI collaborator, they are more likely to devote cognitive resources to optimizing prompts, revising structural elements, and correcting musical details, rather than uncritically accepting the system’s initial outputs. Affectively, the enjoyment and immersion characteristic of the flow state serve as a powerful motivational resource. Learners experiencing flow tend to display heightened exploratory behavior and persistence, which supports sustained, high-intensity engagement during the post-generation refinement phase (Shernoff et al., 2014). On this basis, we hypothesize that creative self-efficacy is positively associated with learning engagement in GenAI-assisted music co-creation (H3) and that flow state is positively associated with learning engagement in GenAI-assisted music co-creation (H4).

This dual-driven engagement not only promotes substantial behavioral involvement but also lays a critical foundation for the development of psychological ownership. While the original Theory of Psychological Ownership (TPO; Pierce et al., 2001) posits three routes to ownership—control over the target, intimate knowing, and investment of self—the present study explicitly narrows its theoretical focus to the latter two. In the context of GenAI-assisted music composition, the mechanical execution and direct “control” over the initial generation are largely delegated to the algorithmic system. Consequently, we argue that for novice learners, psychological ownership is primarily reclaimed and established through compensatory affective and behavioral pathways. In human–computer interaction research, ownership is understood to arise not from physical possession but from the depth of user–system interaction and affective resonance (Kuzminykh and Cauchard, 2020). In music co-creation, the intense concentration and absorption characteristic of flow align closely with the “intimate knowing” route described in TPO (Pierce et al., 2001). As Xu et al. (2024) demonstrate in their study of ownership over human–AI co-creations, users form stronger emotional bonds with AI-generated artifacts when interaction is highly immersive and feedback is perceived as responsive. Flow, therefore, functions not only as a catalyst for sustained creative activity but also as an emotional bridge that connects learners to the emerging musical product. Accordingly, we hypothesize that flow state is positively associated with learners’ psychological ownership of the co-created musical artifact (H5).

Ultimately, the most direct mechanism through which psychological ownership is established lies in the IKEA effect, which is behaviorally expressed through learning engagement. Norton et al. (2012), drawing on behavioral economics experiments, showed that individuals attribute greater subjective value to products into which they have invested substantial labor—a phenomenon summarized as “labor leads to love.” In the context of GenAI-assisted music education, learning engagement—manifested in iterative trial-and-error, parameter adjustment, musical arranging, and detailed polishing following the AI’s initial generation—constitutes the core “investment of self” emphasized in TPO (Avey et al., 2009). Empirical evidence from Xu et al. (2024) further indicates that human-led post-generation refinement is the strongest predictor of the sense that “this is my work” in human–AI collaborations. Building on this line of reasoning, the present study contends that deep engagement during the co-creation process enables learners to internalize the AI-assisted musical product as part of their own psychological domain through the mechanism captured by the IKEA effect. We therefore propose that learning engagement is positively associated with learners’ psychological ownership of the co-created musical artifact (H6).

## 3 Method

### 3.1 Participants

**Figure 2:**
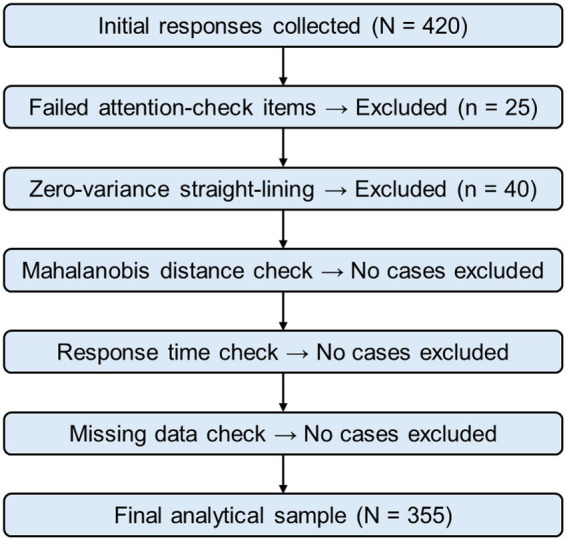
Data screening flowchart.

A total of 420 undergraduate students from eight classes of a music composition course at a comprehensive university completed the online questionnaire. Following a systematic data-screening procedure (described in Section 3.3.2 and illustrated in Figure 2), 65 responses were removed, yielding a final analytical sample of 355 participants. The final sample comprised 200 females (56.3%) and 155 males (43.7%). All eight classes were sections of the same elective music composition course, taught by the same instructor using an identical syllabus, standardized lesson plans, and uniform assignment requirements (i.e., the same text-to-music GenAI tools, the same composition tasks, and the same grading rubric). The classes differed only in scheduled time slots and were held in the same computer-equipped classroom. This instructional uniformity was deliberately maintained to minimize between-class variability in pedagogical delivery, tool usage intensity, and classroom climate. The course was offered as an elective to students from non-music majors. These students align with the purposes of this study as they represent typical novices who face substantial technical barriers in traditional music composition and are therefore well suited for examining the instructional scaffolding effects of text-to-music GenAI tools. Participants came from diverse academic disciplines: STEM (*n* = 123, 34.6%), Arts and Humanities (*n* = 97, 27.3%), Business and Social Sciences (*n* = 84, 23.7%), and other majors (n = 51, 14.4%). The sample was relatively balanced across year levels, comprising 75 first-year (21.1%), 96 s-year (27.0%), 95 third-year (26.8%), and 89 fourth-year students (25.1%). Participants’ ages ranged from 18 to 23 years and older, with the majority (56.1%) aged between 19 and 20.

To characterize baseline proficiency and substantiate their predominantly novice status, we assessed prior music training and GenAI familiarity at the beginning of the course. Most participants reported minimal formal music education: 48.4% had no prior training, and 45.1% had less than 1 year of experience. In addition, 77.2% indicated that they had no previous experience in music composition. With respect to technological familiarity, the sample consisted predominantly of GenAI novices in the auditory domain: 84.2% had never used text-to-music tools before the course, and the remaining 15.8% had only experimented with such tools fewer than three times. Nevertheless, non-trivial minorities within the sample did possess some prior experience: 22.8% reported prior composition experience, 6.5% had 1–3 years of formal music education, and 15.8% had previously used text-to-music tools. The sample is therefore best characterized as predominantly—rather than uniformly—novice in terms of both musical background and GenAI familiarity. This profile supports the assumption that any subsequent cognitive or behavioral changes observed during the study are unlikely to be substantially confounded by prior musical or GenAI expertise. Detailed demographic information is provided in Table 1.

**Table 1:** Demographic information of the participants (*n* = 355).

Basic variable	Category	Frequency	Percentage (%)
Gender	Female	200	56.33
	Male	155	43.67
Age	18	58	16.34
	19	97	27.32
	20	102	28.73
	21	55	15.49
	22	28	7.89
	> = 23	15	4.23
Grade	Year 1	75	21.13
	Year 2	96	27.04
	Year 3	95	26.76
	Year 4	89	25.07
Major	Arts and humanities	97	27.32
	STEM	123	34.65
	Business and social sciences	84	23.66
	Other	51	14.37
Music education experience	without experience	172	48.4
	< 1 year	160	45.1
	1–3 years	23	6.5
Music composition experience	without experience	274	77.18
	with experience	81	22.82
Text-to-music tool use experience	without experience	299	84.23
	less than three times	56	15.77

### 3.2 Instruments

#### 3.2.1 Perceived GenAI support

To assess students’ perceptions of GenAI support in music composition, this study used an adapted version of the Perceived Usefulness scale (Davis, 1989; Hsiao and Tang, 2025; Venkatesh et al., 2012). It is conceptually crucial to distinguish perceived GenAI support, as operationalized in this study, from the traditional perceived usefulness construct found in the Technology Acceptance Model (TAM). While traditional TAM scales primarily measure generic productivity or efficiency beliefs (e.g., “using this system enhances my job performance”), our items were deliberately re-contextualized to capture the extent to which the tool provides *cognitive scaffolding* and *creative support*. Specifically, the items evaluate how the text-to-music tool reduces cognitive barriers and serves as a creative springboard (e.g., “I find the AI music tool useful in supporting my creative ideas”). In the theoretical model, this construct functions as the starting point of the psychological mechanism chain—not merely to predict the intention to adopt the technology, but to quantify the degree to which the AI’s scaffolding frees up learners’ cognitive resources, thereby triggering downstream psychological processes such as creative self-efficacy and flow. Participants rated each of the four adapted items on a 5-point Likert scale ranging from 1 (strongly disagree) to 5 (strongly agree). In the present study, the scale demonstrated good internal consistency, with a Cronbach’s alpha of 0.861.

#### 3.2.2 Creative self-efficacy

To assess learners’ creative self-efficacy in the GenAI-assisted music composition context, this study adapted the Creative Self-Efficacy scale originally developed by Tierney and Farmer (2002). The original measure, designed to capture general creative confidence in organizational settings, was carefully revised to reflect students’ confidence in generating novel musical ideas and addressing compositional challenges in collaboration with the AI tool. The adapted scale included four items, such as “I have confidence in my ability to solve musical composition problems creatively using the AI tool” and “I am confident that I can generate novel musical ideas to guide the AI.” Participants responded on a 5-point Likert scale ranging from 1 (strongly disagree) to 5 (strongly agree). In the present study, the scale demonstrated strong internal consistency, with a Cronbach’s alpha of 0.857.

#### 3.2.3 Flow state

To evaluate learners’ flow state during GenAI-assisted music composition, this study adapted the well-validated Flow State Scale developed by Jackson and Marsh (1996). Originally designed to assess optimal experience in performance contexts, the instrument was carefully contextualized to capture students’ immersive experiences in a human–AI co-creation environment. Items were rephrased to reflect AI-supported music creation while preserving the underlying constructs. Example items include: “I felt challenged by the music creation task, but I believed my skills (with the AI’s help) allowed me to meet the challenge” and “I really enjoyed the experience of creating music with the AI.” Participants indicated their agreement on a 5-point Likert scale ranging from 1 (strongly disagree) to 5 (strongly agree). Construct validity of the adapted scale was examined using confirmatory factor analysis (CFA), and the measurement model demonstrated an excellent fit to the data, *X*^2^ = 39.351, df = 27, X^2^/df = 1.457, *p* > 0.05, CFI = 0.994, TLI = 0.992, RMSEA = 0.036, SRMR = 0.019. The scale also showed excellent internal consistency, with a Cronbach’s alpha of 0.938.

#### 3.2.4 Learning engagement

To evaluate students’ engagement in the GenAI-assisted music composition process, this study adapted the Student Engagement Scale originally developed by Gunuc and Kuzu (2015). Although the original instrument was designed to assess engagement in general educational contexts, it was carefully contextualized to capture students’ involvement during the “generation–modification–refinement” cycle when working with the text-to-music tool. The adapted scale comprises 10 items reflecting three dimensions of engagement: behavioral, cognitive, and emotional (Gunuc and Kuzu, 2015). Item wording was revised to emphasize human–AI collaborative behaviors and iterative musical refinement, while preserving the underlying constructs. Participants indicated their agreement with each item on a 5-point Likert scale ranging from 1 (strongly disagree) to 5 (strongly agree). Construct validity was examined using CFA, and the measurement model showed an excellent fit to the data, *X*^2^ = 41.78, df = 35, X^2^/df = 1.194, *p* > 0.05, CFI = 0.997, TLI = 0.996, RMSEA = 0.023, and SRMR = 0.0181. The scale also demonstrated excellent internal consistency, with an overall Cronbach’s alpha of 0.939.

#### 3.2.5 Psychological ownership

To assess learners’ psychological ownership of the musical artifacts co-created with generative AI, this study adapted the Psychological Ownership Scale originally developed by Avey et al. (2009). Although the original instrument was designed to assess employees’ feelings of possession toward their organizations, it was carefully recontextualized to capture learners’ sense of ownership over human–AI co-created digital artifacts. According to Pierce et al. (2001), the target of psychological ownership can be immaterial creations and ideas; thus, transferring the scale to digital artifacts is theoretically sound. The adapted scale comprises 10 items covering three core dimensions of psychological ownership: identity, belongingness, and accountability. In a student assignment context, “accountability” functions meaningfully, as learners bear the ultimate academic responsibility for the submitted work and must manually audit and correct any AI-generated flaws to meet evaluation standards. Example items include “I feel that if this music is successful, it is my personal success” and “I feel a strong personal connection to this music.”

To empirically validate the dimensional structure of the adapted scale in this novel context, competing measurement models were compared using confirmatory factor analysis (CFA). The hypothesized correlated three-factor model demonstrated an excellent fit to the data (X^2^ = 54.141, df = 32, X^2^/df = 1.692, *p* = 0.009, CFI = 0.992, TLI = 0.988, RMSEA = 0.044, SRMR = 0.019). Simultaneously, a one-factor model—where all 10 items loaded onto a single global latent construct—also yielded an exceptionally strong fit (X^2^ = 59.952, df = 35, X^2^/df = 1.713, *p* = 0.005, CFI = 0.991, TLI = 0.988, RMSEA = 0.045, SRMR = 0.020). While the three-factor structure provided a marginally superior fit, the one-factor model firmly satisfied all recommended stringent criteria (Hu and Bentler, 1999). Consequently, to maintain model parsimony and to align strictly with our theoretical hypotheses (H5, H6)—which posit relationships at the overarching level of psychological ownership rather than at the dimensional level—this construct was operationalized as a unidimensional factor in the subsequent structural equation modeling (SEM). The instrument also showed outstanding internal consistency, with an overall Cronbach’s alpha of 0.948.

#### 3.2.6 Scale translation and adaptation procedures

As described across the instruments above, because the measurement instruments were adapted to fit the context of GenAI-assisted music composition and were administered to Chinese-speaking participants, a rigorous and systematic adaptation protocol was followed (Brislin, 1970). First, to ensure semantic equivalence, all original English items were translated into Chinese using a standard forward-backward translation procedure by two bilingual educational researchers. Second, the items were revised following a principle of minimal necessary modification: general organizational or educational referents were replaced with specific GenAI-assisted co-creation referents, while preserving the underlying theoretical constructs. No items were deleted during this contextualization process. Third, a panel of three experts (two in educational technology and one in music education) reviewed the adapted items for content validity, face validity, and contextual appropriateness, leading to minor wording refinements. Finally, prior to the main study, a pilot test was conducted with 45 undergraduate students (who were excluded from the final sample) to evaluate item clarity, readability, and preliminary internal consistency. All scales demonstrated acceptable reliability (alpha > 0.80) in the pilot phase. The full text of all measurement items is provided in the appendix.

### 3.3 Data collection and analysis

#### 3.3.1 Procedure and data collection

During the course, students were instructed to use text-to-music tools (i.e., Muse AI) to produce initial musical compositions via natural language prompts. They were then required to engage in post-generation refinement by editing and enhancing the AI-generated drafts. Students submitted both the raw AI-generated tracks and their human-edited final versions as course assignments. This pedagogical design situated the data collection within an authentic instructional context in which students engaged in a “generation–modification” loop characteristic of human–AI co-creation. However, it should be noted that the present study assessed students’ perceptions and psychological states associated with this co-creation process through self-report measures, rather than directly analyzing the artifacts or behavioral traces produced during the generation–modification cycle. The ecological contribution of the study therefore lies in the authentic task context in which participants’ responses were situated, rather than in the direct measurement of co-creation behaviors.

Data were collected at the conclusion of the music composition course in collaboration with the instructors. Students were invited to self-report their perceived GenAI support, creative self-efficacy, flow state, learner engagement, and psychological ownership by scanning a QR code that linked to an online questionnaire. The survey required approximately 13 min to complete. Before data collection, all participants were informed that participation was voluntary, their responses would remain anonymous, and the data would be used solely for academic research purposes, thereby ensuring ethical compliance.

#### 3.3.2 Data analysis

Data analysis was conducted using SPSS 30 and AMOS 29, following a two-step quantitative modeling procedure.

Prior to model estimation, a rigorous multi-step data-screening procedure was conducted to ensure data quality (see Figure 2 for a flowchart summary). Of the 420 initial responses, 25 were first removed because respondents failed two embedded attention-check items (e.g., “Please select ‘Strongly Agree’ for this item”). Next, 40 additional responses were excluded on the basis of straight-lining, operationally defined as zero variance across all survey items within a respondent’s record, which indicates non-differentiated responding (Meade and Craig, 2012). The remaining 355 responses were then subjected to further diagnostic checks. Mahalanobis distance (D^2^) values were computed for all cases, and no respondent exceeded the critical χ^2^ threshold at *p* < 0.001, indicating the absence of multivariate outliers (Tabachnick and Fidell, 2019). In addition, response time was examined: the median completion time was approximately 13 min, and no response fell below a minimum plausible threshold of 5 min, suggesting that all retained respondents engaged meaningfully with the survey content. Because the online survey platform required responses to all items, no missing data were present. After all screening steps, the final analytical sample comprised 355 valid responses (retention rate: 84.5%).

To move beyond conventional rules of thumb (e.g., the 10:1 indicator-to-sample ratio or the N ≥ 200 heuristic) and provide a more rigorous justification of sample size adequacy, an RMSEA-based power analysis was conducted following the framework of MacCallum et al. (1996), implemented via the semPower package in R (Moshagen and Erdfelder, 2016). For the hypothesized structural model (df = 623), with *α* = 0.05, a null hypothesis of not-close fit (H₀: RMSEA ≥ 0.05) was tested against the observed model fit. At the obtained sample size of N = 355, the non-centrality parameter (NCP) was 551.36, and the achieved statistical power exceeded 0.9999, indicating a near-certain probability of detecting misspecified models. Furthermore, the minimum sample size required to achieve a conventional power level of 0.80 under the same parameters was estimated at N = 61, confirming that the present sample of 355 participants far surpasses—by approximately a factor of six—the threshold necessary for adequate statistical power. These results provide strong quantitative evidence that the final sample affords more than sufficient statistical power for the planned SEM analyses.

Given that students were nested within eight class sections, the potential for clustering effects was examined prior to model estimation. Intraclass correlation coefficients (ICCs) were computed for all latent construct indicators using one-way random-effects ANOVA, with class membership as the grouping variable. The ICC values across all observed items ranged from 0.002 to 0.038, with a mean ICC of 0.014. All values fell well below the commonly recommended threshold of 0.05 (LeBreton and Senter, 2008), indicating that between-class differences accounted for a negligible proportion of the total variance in the study variables. This result is consistent with the study design, in which all eight sections were taught by the same instructor using a standardized curriculum, identical GenAI tools, and uniform task requirements. These results confirm that the nesting structure did not substantively influence the findings, and a single-level SEM approach was therefore deemed appropriate for the present data.

Subsequently, a CFA was performed to establish the reliability, convergent validity, and discriminant validity of the measurement model encompassing all key constructs. To address the first research question (RQ1), bivariate relationships among these variables were examined using Pearson correlation coefficients derived from the validated measurement model. Furthermore, given that the data were collected via cross-sectional, self-reported questionnaires, Harman’s single-factor test was conducted to assess the potential threat of common-method bias (CMB). All measurement items were entered into an unrotated principal component factor analysis. The results indicated that the first principal component accounted for only 39.0% (i.e., 38.998%) of the total variance, which is well below the recommended threshold of 50% (Podsakoff et al., 2003). Therefore, CMB was not considered a significant concern in the present study.

Subsequently, a CFA was performed to establish the reliability, convergent validity, and discriminant validity of the measurement model encompassing all key constructs. To address the first research question (RQ1), bivariate relationships among these variables were examined using Pearson correlation coefficients derived from the validated measurement model. Furthermore, given that the data were collected via cross-sectional, self-reported questionnaires, two complementary procedures were employed to assess the potential threat of common-method bias (CMB). First, Harman’s single-factor test was conducted**:** all measurement items were entered into an unrotated principal component factor analysis. The results indicated that the first principal component accounted for only 39.0% (i.e., 38.998%) of the total variance, which is well below the recommended threshold of 50% (Podsakoff et al., 2003). Second, an unmeasured latent method factor (ULMF) approach was employed to provide a more stringent post-hoc diagnostic (Podsakoff et al., 2003). A latent common-method variance factor, on which all observed indicators loaded simultaneously, was added to the confirmed multi-factor CFA model. The method factor’s variance was fixed to 1.0, and its covariances with all substantive factors were constrained to zero. Results indicated that the inclusion of the method factor did not substantially improve model fit (ΔCFI = 0.007, below the 0.01 criterion; Cheung and Rensvold, 2002; ΔRMSEA = 0.006; ΔSRMR = 0.003). Moreover, all substantive factor loadings remained significant and highly stable, with a mean absolute change of only 0.025 and a maximum change of 0.082. Taken together, these converging results indicate that CMB does not pose a serious threat to the validity of the present findings.

To address the second research question (RQ2)—which examined the extent to which creative self-efficacy, flow state, and learner engagement serially mediate the relationship between perceived GenAI support and learners’ psychological ownership—structural equation modeling (SEM) was used to test the hypothesized structural model. Importantly, given the one-time, end-of-course cross-sectional design, this analysis was intended to evaluate the statistical consistency of the hypothesized pathways rather than to serve as a formal causal test. In addition, to rigorously assess the significance of the indirect effects, a bias-corrected bootstrapping procedure with 5,000 resamples and 95% confidence intervals (CI) was employed, as this approach provides a robust estimation framework for complex serial mediation models.

Furthermore, although participant background characteristics—including prior music training, prior composition experience, prior text-to-music GenAI tool experience, gender, year level, and academic major—were collected as sample descriptors, they were not included in the baseline structural model because the sample was deliberately recruited from a predominantly novice population with highly restricted variance in domain-relevant prior experience (see Section 3.1). To empirically verify that these background variables did not confound the hypothesized structural relationships, a robustness check was conducted by re-estimating the structural model with all six variables simultaneously entered as covariates. Specifically, prior music training was coded as an ordinal variable (0 = no training, 1 = less than 1 year, 2 = 1–3 years, 3 = more than 3 years), prior composition experience as a binary variable (0 = no, 1 = yes), prior text-to-music GenAI tool experience as a binary variable (0 = never used, 1 = used fewer than three times), gender as a binary variable (0 = male, 1 = female), year level as a continuous variable (1 to 4), and academic major as a set of three dummy-coded indicator variables with STEM as the reference category. All covariates were regressed on every endogenous latent variable in the structural model. The results of these robustness analyses are reported in Section 4.4.

## 4 Results

### 4.1 Confirmatory factor analysis results

Prior to model estimation, the distributional properties of all observed variables were examined. Univariate skewness values ranged from −0.255 to 0.037 and kurtosis values ranged from −1.271 to −0.975, all well within the recommended thresholds of |skewness| < 2 and |kurtosis| < 7 (Kline, 2023). Mardia’s multivariate kurtosis coefficient was 40.020 (c.r. = 7.018), indicating some departure from multivariate normality. To address this, the Bollen-Stine bootstrap procedure with 2,000 resamples was employed to verify model fit (Bollen and Stine, 1992).

A CFA was conducted to evaluate the measurement model. The model demonstrated an excellent fit to the data: χ^2^(619) = 625.71, χ^2^/df = 1.01, CFI = 0.993, TLI = 0.991, RMSEA = 0.006, and SRMR = 0.029. All indices met or exceeded commonly accepted criteria for excellent model fit (Hu and Bentler, 1999), namely χ^2^/df < 3, CFI and TLI > 0.95, RMSEA < 0.06, and SRMR < 0.08. The Bollen-Stine bootstrap *p* value was 0.824, confirming that the model fit remained adequate under non-normal distributional conditions.

Regarding model specification, no modification indices were consulted at any stage of the analysis, and no correlated error terms were added in the measurement model. All parameter specifications were derived strictly from theoretical considerations and prior empirical literature. Inspection of the standardized residual covariance matrix revealed that no standardized residual exceeded |2.58|, with the largest absolute value being 1.878, well below the conventional threshold (Byrne, 2013). This indicates that the hypothesized measurement model adequately reproduced the observed covariance patterns without systematic areas of localized misfit. The favorable fit indices are therefore attributable to the use of well-validated scales drawn from the existing literature, a theoretically grounded model specification without any *post hoc* modifications, and a relatively homogeneous sample of university music students who completed the same AI-assisted composition task.

As shown in Table 2, all factor loadings were statistically significant (*p* < 0.001) and substantial in magnitude, with standardized estimates above the recommended threshold of 0.60 (Mardia et al., 2024). This indicates that all items functioned as strong indicators of their respective latent constructs.

**Table 2:** Unstandardized and standardized estimates in the CFA model.

Variables	Directions	Variables	Standardized estimates	Unstandardized estimates	S.E.	C.R.	*p*
GenAIsupport1	<−--	GenAI Support	0.788	1			
GenAIsupport2	<−--	GenAI Support	0.767	0.983	0.067	14.571	***
GenAIsupport3	<−--	GenAI Support	0.776	1.034	0.071	14.602	***
GenAIsupport4	<−--	GenAIsupport	0.788	1.031	0.069	15.024	***
Self-efficacy1	<−--	Creative Self-efficacy	0.786	1			
Self-efficacy2	<−--	Creative Self-efficacy	0.75	0.936	0.066	14.269	***
Self-efficacy3	<−--	Creative Self-efficacy	0.777	0.997	0.07	14.324	***
Self-efficacy4	<−--	Creative Self-efficacy	0.782	0.982	0.066	14.791	***
Flowstate1	<−--	Flows State	0.791	1			
Flowstate2	<−--	Flows State	0.801	0.957	0.057	16.838	***
Flowstate3	<−--	Flows State	0.804	1.004	0.059	16.902	***
Flowstate4	<−--	Flows State	0.772	0.954	0.059	16.047	***
Flowstate5	<−--	Flows State	0.812	1.019	0.06	17.084	***
Flowstate6	<−--	Flows State	0.791	0.994	0.06	16.522	***
Flowstate7	<−--	Flows State	0.814	1.037	0.06	17.14	***
Flowstate8	<−--	Flows State	0.774	0.93	0.058	16.091	***
Flowstate9	<−--	Flows State	0.767	0.944	0.059	15.91	***
Engagement1	<−--	Engagement	0.784	1			
Engagement2	<−--	Engagement	0.767	1	0.063	15.746	***
Engagement3	<−--	Engagement	0.811	1.066	0.063	17.005	***
Engagement4	<−--	Engagement	0.755	1.004	0.065	15.39	***
Engagement5	<−--	Engagement	0.754	0.987	0.064	15.363	***
Engagement6	<−--	Engagement	0.79	1.049	0.064	16.395	***
Engagement7	<−--	Engagement	0.781	1.028	0.064	16.075	***
Engagement8	<−--	Engagement	0.788	1.032	0.063	16.305	***
Engagement9	<−--	Engagement	0.772	1.015	0.064	15.829	***
Engagement10	<−--	Engagement	0.786	1.063	0.066	16.162	***
Ownership1	<−--	Ownership	0.776	1			
Ownership2	<−--	Ownership	0.829	1.117	0.065	17.315	***
Ownership3	<−--	Ownership	0.814	1.093	0.065	16.773	***
Ownership4	<−--	Ownership	0.808	1.083	0.065	16.711	***
Ownership5	<−--	Ownership	0.841	1.115	0.064	17.542	***
Ownership6	<−--	Ownership	0.804	1.06	0.064	16.626	***
Ownership7	<−--	Ownership	0.78	0.99	0.062	15.946	***
Ownership8	<−--	Ownership	0.784	1.03	0.064	16.101	***
Ownership9	<−--	Ownership	0.791	1.02	0.063	16.256	***
Ownership10	<−--	Ownership	0.799	1.083	0.066	16.47	***

***p < 0.001.

The composite reliability (CR) and convergent validity of the constructs were then examined. As reported in Table 3, the CR values for psychological ownership, learner engagement, flow state, creative self-efficacy, and perceived GenAI support were 0.948, 0.939, 0.938, 0.857, and 0.861, respectively. All values exceeded the recommended minimum of 0.70, indicating satisfactory internal consistency (Hair et al., 2010). The average variance extracted (AVE) values for these constructs were 0.645, 0.607, 0.627, 0.599, and 0.608, respectively. All AVE values were above the benchmark of 0.50, providing strong evidence of convergent validity and confirming that each construct accounted for a substantial proportion of variance in its indicators (Hair et al., 2010).

**Table 3:** Reliability and validity of the constructs and their correlation.

Variables	CR	AVE	Ownership	Engagement	Flow State	Creative Self-efficacy	GenAI Support
Ownership	0.948	0.645	0.803				
Engagement	0.939	0.607	0.518***	0.779			
Flow State	0.938	0.627	0.507***	0.488***	0.792		
Creative Self-efficacy	0.857	0.599	0.520***	0.480***	0.462***	0.774	
GenAI Support	0.861	0.608	0.486***	0.426***	0.431***	0.504***	0.780

***p < 0.001.

Discriminant validity was also supported. Following the Fornell–Larcker criterion, the square root of the AVE for each construct (displayed on the diagonal of the correlation matrix and ranging from 0.774 to 0.803) exceeded its correlations with all other constructs (which ranged from 0.426 to 0.520). This pattern indicates that each construct shared more variance with its own indicators than with any other construct in the model, thereby satisfying the criteria for discriminant validity. To further rigorously assess the discriminant validity—especially given the conceptual proximity between flow and engagement—we computed the Heterotrait-Monotrait (HTMT) ratio. The HTMT value between flow state and learner engagement was 0.488, well below the stringent threshold of 0.85, confirming their empirical distinctiveness.

Furthermore, a competing measurement model analysis was conducted. The baseline five-factor model [χ^2^ (619) = 625.71, CFI = 0.993, RMSEA = 0.006] was compared against an alternative four-factor model where all items for flow state and learner engagement were constrained to load onto a single latent factor. The alternative model yielded a significantly poorer fit to the data [χ^2^ (623) = 2146.991, χ^2^/df = 3.446, CFI = 0.826, TLI = 0.814, RMSEA = 0.083, SRMR = 0.085]. A chi-square difference test confirmed that the baseline model was significantly superior, justifying the treatment of flow and engagement as distinct constructs.

### 4.2 Construct relationship (RQ1)

The correlation matrix in Table 3 reveals a conceptually coherent pattern of associations among the study variables. Perceived GenAI support was significantly and positively correlated with creative self-efficacy (*r* = 0.504, *p* < 0.001), flow state (*r* = 0.431, *p* < 0.001), and learner engagement (*r* = 0.426, *p* < 0.001). In turn, these cognitive, affective, and behavioral variables were all positively associated with psychological ownership (creative self-efficacy: *r* = 0.520, *p* < 0.001; flow state: *r* = 0.507, *p* < 0.001; learner engagement: *r* = 0.518, *p* < 0.001). Perceived GenAI support also showed a significant positive correlation with psychological ownership (*r* = 0.486, *p* < 0.001).

Overall, this pattern of consistently positive correlations among the technological antecedent (perceived GenAI support), the intermediate learning experiences (creative self-efficacy, flow, and engagement), and the outcome variable (psychological ownership) aligns well with the proposed nomological network. Higher levels of perceived AI support and deeper psychological involvement in the music composition process were associated with stronger feelings of ownership over the co-created digital artifacts.

### 4.3 Structural equation modeling and serial mediation analysis results (RQ2)

The SEM was employed to test the hypothesized direct associations among the constructs (H1–H6). The structural model (Figure 1) showed excellent fit: χ^2^(623) = 685.08, χ^2^/df = 1.10, CFI = 0.993, TLI = 0.992, RMSEA = 0.017, and SRMR = 0.068. All indices satisfied the recommended thresholds for excellent fit (Hu and Bentler, 1999). Table 4 and Figure 2 present the standardized path coefficients.

**Table 4:** Results of regression analysis with SEM.

Hypotheses	Variables	Directions	Variables	Standardized	Unstandarzied				Supported (Yes/No)
				Estimate	Estimate	S.E.	C.R.	*P*	
H1	Creative Self-efficacy	<−--	GenAI Support	0.533	0.546	0.065	8.395	***	Yes
H2	Flow State	<−--	Creative Self-efficacy	0.488	0.505	0.063	8.062	***	Yes
H3	Engagement	<−--	Creative Self-efficacy	0.35	0.342	0.061	5.591	***	Yes
H4	Engagement	<−--	Flow State	0.317	0.299	0.056	5.328	***	Yes
H5	Ownership	<−--	Flow State	0.337	0.335	0.057	5.859	***	Yes
H6	Ownership	<−--	Engagement	0.359	0.378	0.061	6.184	***	Yes

The results indicate that all four hypothesized predictors—perceived GenAI support, creative self-efficacy, flow state, and learner engagement—were significantly and positively associated with psychological ownership. Specifically, perceived GenAI support was positively related to creative self-efficacy (*β* = 0.533, *p* < 0.001), supporting H1. Creative self-efficacy, in turn, was positively associated with both flow state (*β* = 0.488, *p* < 0.001) and learner engagement (*β* = 0.350, *p* < 0.001), supporting H2 and H3. Flow state during the AI-assisted composition process was positively related to learner engagement (*β* = 0.317, *p* < 0.001), supporting H4.

**Figure 3:**
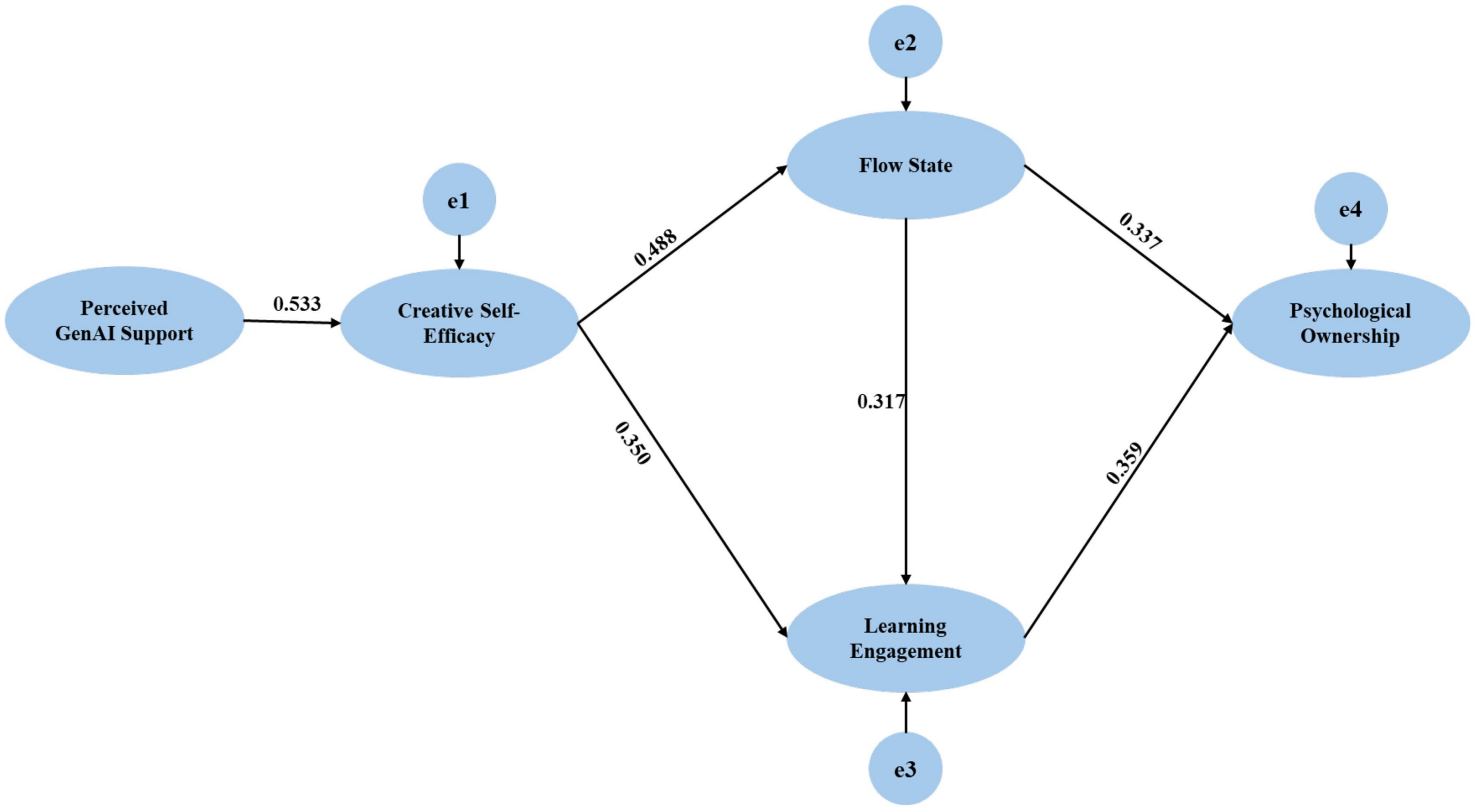
Structural equation model with standardized regression weights.

With respect to the outcome variable, both flow state (*β* = 0.337, *p* < 0.001) and learner engagement (*β* = 0.359, *p* < 0.001) were positively related to psychological ownership of the co-created musical artifacts, thereby supporting H5 and H6. Taken together, these findings indicate a coherent pattern of direct associations linking perceived GenAI support to psychological ownership via learners’ creative self-efficacy, flow experiences, and engagement (Figure 3).

To further validate the hypothesized full mediation structure, we compared the baseline model (Model 1) with an alternative partial mediation model (Model 2). Model 2 included two additional direct paths: one from perceived GenAI support to psychological ownership and one from creative self-efficacy to psychological ownership. These paths tested whether the effects of perceived GenAI support and creative self-efficacy on psychological ownership are fully mediated by flow state and learner engagement, or whether significant direct associations remain. The chi-square difference test indicated that the partial mediation model did not significantly improve model fit, Δχ^2^(2) = 4.04, *p* = 0.133. The change in CFI was negligible (ΔCFI < 0.001), well below the 0.01 threshold recommended by Cheung and Rensvold (2002). Moreover, BIC favored the more parsimonious full mediation model (Model 1: BIC = 1154.85; Model 2: BIC = 1162.55). These results suggest that the effects of perceived GenAI support and creative self-efficacy on psychological ownership are fully transmitted through flow state and learner engagement, and the full mediation model was therefore retained as the final structural model.

To address RQ2 regarding the serial mediating roles of creative self-efficacy, flow state, and learner engagement, a bias-corrected bootstrapping procedure with 5,000 resamples was conducted within the SEM framework using AMOS user-defined estimands to estimate specific indirect effects (Gaskin, 2016; Preacher and Hayes, 2008). Unstandardized indirect effects were computed as the product of the constituent unstandardized path coefficients along each pathway, following the recommendations of Hayes (2017). This analysis was intended to clarify the sequential associations between the technological antecedent and psychological ownership rather than to make causal claims.

As reported in Table 5, perceived GenAI support was positively related to psychological ownership through three significant specific indirect pathways. The total indirect effect of perceived GenAI support on psychological ownership was statistically significant (*β* = 0.194, 95% BC CI [0.130, 0.268]); zero did not fall within the 95% bias-corrected confidence interval, indicating a significant overall mediating effect of the intermediate psychological variables.

**Table 5:** Bootstrapping results for the standardized indirect relationships (Mediation analysis).

Indirect Pathway	Estimate (β)	Boot LLCI	Boot ULCI	p
Ind1: GenAI Support → CSE → Flow State → Ownership	0.092	0.052	0.145	***
Ind2: GenAI Support → CSE → Engagement → Ownership	0.071	0.038	0.120	***
Ind3: GenAI Support → CSE → Flow State → Engagement → Ownership	0.031	0.018	0.051	***
Total Indirect Effect: GenAI Support → … → Ownership	0.194	0.130	0.268	***

Estimate = Unstandardized indirect effect (product of unstandardized path coefficients along each pathway); Boot LLCI = lower limit of the 95% bias-corrected confidence interval; Boot ULCI = upper limit of the 95% bias-corrected confidence interval. Bootstrap sample size = 5,000. An indirect effect is considered significant when the 95% BC CI does not contain zero. CSE = Creative self-efficacy. Ind1 represents the serial mediation through creative self-efficacy and flow state; Ind2 represents the serial mediation through creative self-efficacy and learner engagement; Ind3 represents the full serial chain through all three mediators.

Examining the three specific indirect pathways, the strongest was the serial mediation through creative self-efficacy and flow state (Ind1: β = 0.092, 95% BC CI [0.052, 0.145]), suggesting that perceived GenAI support is associated with higher creative self-efficacy, which in turn is linked to deeper flow experiences and, subsequently, a greater sense of psychological ownership. The second pathway operated through creative self-efficacy and learner engagement (Ind2: *β* = 0.071, 95% BC CI [0.038, 0.120]), indicating that the enhanced creative self-efficacy associated with GenAI support is also related to greater engagement, which is further linked to psychological ownership. The third pathway represented the full serial chain through all three mediators—creative self-efficacy, flow state, and learner engagement (Ind3: β = 0.031, 95% BC CI [0.018, 0.051])—providing evidence for a sequential process in which creative self-efficacy is linked to flow state, flow state to engagement, and engagement to psychological ownership.

Notably, although all three specific indirect pathways were significant, the pathway involving flow state (Ind1) carried the largest indirect effect, suggesting that flow experience may play a particularly important role in linking creative self-efficacy to psychological ownership in AI-assisted music composition contexts.

In combination with the fully supported direct paths (H1–H6), these bootstrapping results provide strong empirical support for the proposed serial mediation model. The findings suggest that perceived GenAI support is linked to higher creative self-efficacy, which is associated with more intense flow experiences and greater engagement; together, these psychological processes are related to a heightened sense of ownership over AI-assisted musical compositions.

### 4.4 Robustness checks: controlling for background characteristics

To rule out the possibility that the hypothesized structural paths were confounded by individual differences in prior experience or demographic characteristics, the structural model was re-estimated with six covariates simultaneously regressed on all endogenous latent variables: prior music training, prior composition experience, prior text-to-music GenAI tool experience, gender, year level, and academic major (dummy-coded). The covariate effects were uniformly small and non-significant: standardized coefficients ranged from −0.04 to 0.07, and none reached statistical significance at the 0.05 level. Prior music training did not significantly predict creative self-efficacy (*β* = 0.04, *p* = 0.438), flow state (*β* = 0.02, *p* = 0.714), learner engagement (*β* = 0.05, *p* = 0.347), or psychological ownership (*β* = 0.03, *p* = 0.592). Similarly, prior composition experience showed no significant effects on any endogenous variable (*β* values ranging from 0.03 to 0.06, all *p-*values > 0.25). Prior text-to-music GenAI tool experience exhibited the largest—but still non-significant—effect on creative self-efficacy (*β* = 0.07, *p* = 0.182), with all other paths remaining negligible (*β* values ≤ 0.05, all *p-*values > 0.30). Gender, year level, and academic major likewise showed no significant associations with any endogenous construct (all *p-*values > 0.40).

Critically, the inclusion of these covariates produced negligible changes in the hypothesized structural paths. All previously significant paths remained significant at the same level, all previously non-significant paths remained non-significant, and the direction of every coefficient was preserved. The largest absolute change in any standardized path coefficient was 0.013, and the mean absolute change across all structural paths was 0.006. These results confirm that the theoretical relationships identified in the baseline model are robust and not attributable to confounding effects of prior musical training, composition experience, GenAI familiarity, or demographic background. The negligible influence of these covariates is consistent with the composition of the present sample: 93.5% of participants reported less than 1 year of formal music training or none at all, 77.2% had no prior composition experience, and 84.2% had never used text-to-music GenAI tools, leaving highly restricted variance in these background variables.

## 5 Discussion

The primary objective of this study was to open the “black box” of human–AI co-creation by examining how predominantly novice learners develop a sense of psychological ownership over musical artifacts generated and refined with the assistance of GenAI. The recent emergence of text-to-music systems has substantially lowered the technical barriers to music composition, but it has also raised pedagogical concerns about the possible erosion of human creative agency and the risk that learners may become passive prompt engineers (Baird and Maruping, 2021; Weisz et al., 2023). To address this issue, we implemented a pedagogical design centered on a “generation–modification” loop with 355 non–music-major undergraduates. Drawing on TPO, we tested a theory-driven sequential mediation model of associations that clarifies the cognitive, affective, and behavioral processes that are statistically consistent with the link between technological scaffolding and a sense of personal appropriation. The findings show that perceived GenAI support is not negatively linked to human agency; when thoughtfully embedded in the learning workflow, it is instead associated with higher creative self-efficacy, flow, and learner engagement, which together underpin psychological ownership.

### 5.1 Discussion of key findings

The first key finding is that perceived GenAI support is significantly and positively associated with learners’ creative self-efficacy (H1). This suggests that the scaffolding provided by text-to-music tools may function as a cognitive springboard, potentially alleviating the initial anxiety and cognitive load associated with the blank page that predominantly novices often face. This result is consistent with Bandura’s (1997) social cognitive theory, which identifies mastery experiences and persuasive feedback (including technologically mediated forms) as central sources of self-efficacy. It also converges with recent work on AI-assisted creativity (e.g., Weisz et al., 2023), which shows that generative models are associated with bolstered creative confidence by supplying competent initial drafts. At the same time, our findings extend beyond traditional technology acceptance perspectives (e.g., Crompton and Burke, 2023), which tend to conceptualize AI primarily as an efficiency-enhancing tool. Interpreted through the lens of cognitive offloading, GenAI in music composition is associated with a shift of lower-level technical execution (e.g., chord progressions, instrumentation choices) to the system (Weisz et al., 2023). In line with Cognitive Load Theory (Sweller, 1988) and research on strategic cognitive offloading (Risko and Gilbert, 2016), this redistribution of effort is statistically consistent with the freeing of limited working-memory resources, enabling learners to concentrate on higher-order evaluative and aesthetic decisions. In doing so, it is related to a stronger belief that they can successfully complete the composition task.

The results further show that creative self-efficacy is positively related to both flow state (H2) and learner engagement (H3), and that flow is, in turn, associated with deeper engagement (H4). This pattern indicates that confidence in one’s creative capacity is a central precondition for entering an optimal state of psychological immersion and for sustaining behavioral effort in human–AI collaboration. These findings align closely with Csikszentmihalyi’s (2014) Flow Theory and Pekrun’s (2006) Control–Value Theory of Achievement Emotions. According to Pekrun, learners who experience a strong sense of control (high self-efficacy) over a valued activity are more likely to report positive activating emotions, such as enjoyment and flow. In the present context, GenAI was associated with elevated perceived baseline abilities, which brought the challenge of editing and refining the AI-generated music into balance with their enhanced skills. This equilibrium was linked to reduced frustration and higher reported flow. In line with Fredricks et al. (2004), who conceptualize engagement as a multidimensional construct shaped by affective experiences, our findings also help explain the relationship between creative agency and the iterative modification process. The intrinsic rewards associated with flow, combined with a strong sense of creative agency, are associated with learners to invest effort in iteratively modifying and improving the music rather than remaining passive consumers of AI-generated content.

The most consequential findings address RQ2, demonstrating that flow state and learner engagement not only show significant positive associations with psychological ownership (H5, H6), but also jointly serve as key serial mediators characterizing the indirect pathways between perceived GenAI support and ownership. The decomposition of the total indirect effect into three specific pathways (Preacher and Hayes, 2008) reveals a nuanced picture of how this process unfolds. The strongest specific indirect pathway operated through creative self-efficacy and flow state (Ind1: *B* = 0.092, 95% BC CI [0.052, 0.145]), followed by the pathway through creative self-efficacy and learner engagement (Ind2: *B* = 0.071, 95% BC CI [0.038, 0.120]), and the full serial chain through creative self-efficacy, flow state, and learner engagement (Ind3: *B* = 0.031, 95% BC CI [0.018, 0.051]). These findings indicate that technological assistance alone is statistically insufficient to be associated with a sense of “mine-ness.” Instead, musical artifacts are more likely to be psychologically owned when learners invest substantial emotional and behavioral resources in their refinement. This result is strongly compatible with the core tenets of TPO (Pierce et al., 2001) and offers empirical support for the IKEA effect (Norton et al., 2012) in a digital, AI-mediated creative context.

The relative magnitude of the three indirect effects offers further theoretical insight. The finding that the flow-mediated pathway (Ind1) carried the largest indirect effect underscores the primacy of affective immersion in cultivating ownership over AI-assisted creative outputs. This suggests that, in the context of human–AI co-creation, the deep absorption and emotional resonance characteristic of flow may be a more potent mechanism for fostering psychological ownership than behavioral engagement alone (Ind2). Notably, the full serial chain (Ind3), although the smallest in magnitude, was nonetheless statistically significant, providing evidence for a sequential process in which creative self-efficacy facilitates flow, flow deepens engagement, and engagement in turn strengthens ownership. This stepwise transmission is consistent with Csikszentmihalyi (2014) theorizing that flow serves as a gateway to sustained involvement, and extends it by demonstrating that this cascading process ultimately culminates in a sense of personal possession over the creative product.

Prior research on automation often warns that extensive machine intervention is often associated with user alienation and diminished ownership (Baird and Maruping, 2021). Our findings complicate this view. The divergence can be traced to the specific pedagogical structure of the generation–modification loop. By requiring students to edit, evaluate, and enhance the AI’s initial output, the design was linked to an “investment of self.” As reflected in the specific indirect effects, flow was associated with the strongest pathway to ownership (Ind1), consistent with the interpretation that affective investment through generating an emotional bond with the evolving composition constitutes the primary mechanism, while sustained engagement reflected the association with labor investment through repeated, effortful refinement via a somewhat smaller but still significant pathway (Ind2). Under these conditions, GenAI was not experienced as an autonomous creator displacing the learner, but as a collaborative partner embedded within the creative process. It is precisely the human effort statistically linked to the post-AI generation phase that appears to shift the locus of ownership from the machine back to the learner.

### 5.2 Implications, limitations, and future research

#### 5.2.1 Theoretical implications

This study makes two main theoretical contributions to research on educational technology and human–computer interaction.

First, it extends TPO (Pierce et al., 2001) to the context of GenAI-supported learning by identifying a mechanism of ownership formation that is structurally distinct from that observed with traditional digital music tools. In conventional computer-assisted composition environments (e.g., DAWs such as GarageBand or FL Studio), the learner retains full authorial agency from the outset: every note, rhythm, and arrangement originates from the learner’s deliberate action, and psychological ownership accumulates progressively as effort is invested. GenAI-assisted composition fundamentally disrupts this accumulation logic. Because the system autonomously generates a complete musical artifact from a brief textual prompt, the learner’s initial creative contribution is linguistic and curatorial rather than musical and constructive. This agency delegation creates what we term an ownership gap—a psychological deficit that arises when a substantive creative product exists but the human contributor cannot straightforwardly attribute its genesis to personal effort or skill. Our findings suggest that this gap is not permanent; rather, it is closed through a compensatory reclamation process in which the learner invests subsequent editorial labor—evaluating, selecting, revising prompts, and iteratively refining AI-generated outputs—that progressively re-anchors the artifact to the self. This reclamation mechanism reframes the IKEA effect (Norton et al., 2012) in a theoretically important way. The original IKEA effect describes valuation gains from assembling pre-designed components; in GenAI co-creation, the operative effort is not constructive assembly but curatorial editing—a qualitatively different form of labor that involves aesthetic judgment, selective retention, and iterative prompt refinement applied to machine-generated material. We therefore propose the concept of a curatorial IKEA effect to capture this distinct pathway: ownership emerges not because learners build an artifact from raw materials, but because they exercise sustained evaluative agency over an artifact whose initial form was delegated to AI. This distinction has non-trivial theoretical implications, because it suggests that the psychological routes through which effort translates into ownership differ depending on whether the human role is primarily generative (as with traditional tools) or primarily editorial (as with GenAI), even when the final level of perceived ownership is comparable.

Second, the study helps shift the theoretical conversation beyond traditional technology acceptance models (e.g., TAM), which emphasize perceived usefulness and ease of use (Crompton and Burke, 2023), toward a focus on deeper psychological and emotional appropriation. By validating a serial mediation model, we open up the “black box” of human–AI collaboration and demonstrate that the association between perceived technological support and psychological ownership operates through a sequential cognitive–affective–behavioral process (creative self-efficacy → flow state → learner engagement). Crucially, the GenAI context does not merely provide a new empirical setting for this classic mediational chain; it qualitatively transforms the psychological content of each mediating construct. In traditional composition learning, creative self-efficacy refers to the learner’s belief in their capacity to generate musical ideas from domain-specific knowledge. In GenAI-assisted composition, the referent of self-efficacy shifts toward the learner’s confidence in *directing, evaluating, and curating* AI output—a metacognitive and editorial form of efficacy that presupposes a fundamentally different competence profile. Similarly, the flow state in traditional composition is sustained by the intrinsic challenge of translating musical ideas into sound; in GenAI-assisted composition, flow is triggered and maintained by a rapid prompt–output–evaluation–refinement cycle whose temporal dynamics are qualitatively distinct: the near-instantaneous generation of a complete artifact compresses the gap between intention and feedback, creating a low-friction iterative loop that may lower the skill threshold for entering flow while simultaneously restructuring its challenge–skill balance. Learner engagement, in turn, is sustained not by the progressive construction of an artifact but by the iterative refinement of one—a mode of behavioral investment that is evaluative and convergent rather than generative and divergent. These transformations imply that the serial mediation pathway identified in this study, while formally analogous to pathways documented in traditional learning environments, is substantively novel in its underlying psychological mechanisms.

Third, the findings reveal that the low-friction prompt-to-product generation afforded by GenAI creates a distinctive structural condition for ownership formation that has no direct parallel in prior educational technology research. Traditional music composition presents high entry barriers—proficiency in music theory, instrument technique, and notation systems—that make the initial production of a complete artifact effortful and slow. GenAI collapses these barriers: a single textual prompt can yield a fully orchestrated piece in seconds. This radical compression of the production cycle has a paradoxical implication for psychological ownership. On the one hand, the ease of initial generation should, according to effort-based accounts of ownership (Baird and Maruping, 2021), reduce ownership because minimal effort is invested. On the other hand, our data show that ownership levels were robust, suggesting that the low-friction generation serves a scaffolding function: by providing an externalized creative starting point, it enables learners who lack domain-specific skills to enter the evaluative–refinement cycle that ultimately drives ownership. In effect, GenAI does not eliminate the effort required for ownership but redistributes it from the generative phase (where novices face prohibitive skill barriers) to the curatorial phase (where the requisite competencies—aesthetic judgment, prompt literacy, iterative comparison—are more accessible to non-experts). This redistribution mechanism offers a theoretically generative framework for understanding ownership formation across a broader class of human–AI co-creation scenarios, including AI-assisted writing, visual art generation, and code co-authoring, where similar prompt-to-product dynamics obtain.

#### 5.2.2 Practical implications

The findings also yield several practical implications for educators and developers of educational technologies.

For instructional designers and teachers, the results underscore the importance of embedding a structured “generation–modification” loop within AI-assisted learning activities. Tasks that permit one-click generation followed by immediate submission are unlikely to foster psychological ownership or deep learning. Instead, assessment criteria and task instructions should explicitly require students to critique, edit, and iteratively refine AI-generated drafts. This deliberate human-in-the-loop design creates productive friction that prompts learners to contribute their own ideas and labor, transforming an AI-produced draft into a personally meaningful artifact.

For developers of generative AI tools in education, the findings point to the value of designing systems as collaborative partners rather than autonomous “oracles” (Weisz et al., 2023). Interfaces should afford fine-grained user control, transparent access to intermediate versions, and rich options for iterative adjustment. Features that make it easy to compare, modify, and layer AI suggestions with learners’ own contributions are likely to sustain flow, support creative self-efficacy, and, ultimately, strengthen users’ sense of ownership over the outcomes.

#### 5.2.3 Limitations and future research

Despite the insightful implications, several limitations should be acknowledged, each of which suggests directions for future work. First, the cross-sectional design and the reliance on a single-course setting limit the ability to draw strong causal conclusions about the relationships among GenAI support, self-efficacy, flow, engagement, and psychological ownership. Longitudinal or experimental designs conducted across diverse educational contexts would allow researchers to track how these constructs co-develop over time as learners gain experience with AI-supported creation. Second, the study relies primarily on self-report measures, which are susceptible to common-method bias and subjective interpretation. Although the course required students to submit both AI-generated original tracks and human-edited final versions—thereby embedding data collection within an authentic co-creation workflow—the core analytical variables were end-of-course perceptual reports rather than objective behavioral indicators derived from the creative process itself. The study did not capture process-level data such as the number of revisions, editing duration, number of generated versions, prompt iteration logs, or systematic comparisons between AI-generated originals and human-revised outputs. Incorporating such objective behavioral metrics in future research would provide more direct evidence of how learners actually interact with GenAI tools during the generation–modification cycle and would substantially strengthen the ecological validity of the findings. More broadly, future research could triangulate survey data with multimodal learning analytics—for example, system log files to capture actual patterns of editing and interaction, or physiological and behavioral indicators (e.g., eye-tracking, EEG, or fine-grained interaction traces) to provide more direct evidence of flow and engagement during human–AI collaboration. Third, although robustness checks confirmed that prior music training, composition experience, and GenAI familiarity did not significantly affect the structural paths in the present sample, this null finding is partly attributable to the restricted variance in these background variables among the predominantly novice participants. Future research recruiting samples with greater heterogeneity in domain expertise and GenAI experience—for example, by comparing predominant novices with musically trained students or experienced GenAI users—should model these variables as substantive predictors or moderators rather than mere covariates, as their influence may become meaningful in more heterogeneous populations. Finally, while the present study adapted a perceived usefulness scale to capture GenAI support, future research should develop and validate a dedicated perceived creative scaffolding instrument to ensure robust discriminant validity from traditional technology acceptance constructs.

## Data Availability

The raw data supporting the conclusions of this article will be made available by the authors, without undue reservation.
